# Impact of cryopreservation on CAR T production and clinical response

**DOI:** 10.3389/fonc.2022.1024362

**Published:** 2022-10-06

**Authors:** Karin Brezinger-Dayan, Orit Itzhaki, Jenny Melnichenko, Adva Kubi, Li-at Zeltzer, Elad Jacoby, Abraham Avigdor, Ronnie Shapira Frommer, Michal J. Besser

**Affiliations:** ^1^ Ella Lemelbaum Institute for Immuno Oncology, Sheba Medical Center, Ramat Gan, Israel; ^2^ Division of Pediatric Hematology and Oncology, The Edmond and Lily Safra Children’s Hospital, Sheba Medical Center, Ramat Gan, Israel; ^3^ Department of Hematology, Sackler School of Medicine, Tel Aviv University, Tel Aviv, Israel; ^4^ Department of Bone Marrow Transplantation, Sheba Medical Center, Ramat Gan, Israel; ^5^ Davidoff Center, Rabin Medical Center, Petah Tikva, Israel; ^6^ Department of Clinical Microbiology and Immunology, Sackler School of Medicine, Tel Aviv University, Tel Aviv, Israel

**Keywords:** CAR T cells, cryopreservation, PBMC, CAR T manufacturing, clinical trial

## Abstract

Adoptive cell therapy with chimeric antigen receptor (CAR) T cells has become an efficient treatment option for patients with hematological malignancies. FDA approved CAR T products are manufactured in centralized facilities from fresh or frozen leukapheresis and the cryopreserved CAR T infusion product is shipped back to the patient. An increasing number of clinical centers produce CAR T cells on-site, which enables the use of fresh and cryopreserved PBMCs and CAR T cells. Here we determined the effect of cryopreservation on PBMCs and CD19 CAR T cells in a cohort of 118 patients treated with fresh CAR T cells and in several patients head-to-head. Cryopreserved PBMCs, obtained from leukapheresis products, contained less erythrocytes and T cells, but were sufficient to produce CAR T cells for therapy. There was no correlation between the recovery of PBMCs and the transduction efficacy, the number of CAR T cells obtained by the end of the manufacturing process, the *in vitro* reactivity, or the response rate to CAR T therapy. We could show that CAR T cells cryopreserved during the manufacturing process, stored and resumed expansion at a later time point, yielded sufficient cell numbers for treatment and led to complete remissions. Phenotype analysis including T cell subtypes, chemokine receptor and co-inhibitory/stimulatory molecules, revealed that fresh CAR T cells expressed significantly more TIM-3 and contained less effector T cells in comparison to their frozen counterparts. In addition, fresh CAR T infusion products demonstrated increased *in vitro* anti-tumor reactivity, however cryopreserved CAR T cells still showed high anti-tumor potency and specificity. The recovery of cryopreserved CAR T cells was similar in responding and non-responding patients. Although fresh CAR T infusion products exhibit higher anti-tumor reactivity, the use of frozen PBMCs as staring material and frozen CAR T infusion products seems a viable option, as frozen products still exhibit high *in vitro* potency and cryopreservation did not seem to affect the clinical outcome.

## Introduction

Clinical success of adoptive cell therapy with chimeric antigen receptor T cells (CAR T) has led to FDA approval of several CAR T therapies in patients with relapsed or refractory (r/r) hematological malignancies (1–4).

The production of CAR T cells for therapy is comprised of multiple steps: First, peripheral blood mononuclear cells (PBMC) are isolated from patients’ leukapheresis products. Then, PBMCs or isolated, purified T cells are activated, transduced with the CAR transgene and further expanded to the required cell number for therapy. After quality control (QC) testing, the CAR product is intravenously administered to the patient. Typically, patients undergo lymphodepleting conditioning prior to cell infusion.

The number of clinical centers initiating trials with in-house produced CAR T is on a constant rise. To date over 500 active clinical trials with CAR T cells are registered, about half of which utilize CAR T against the CD19 antigen (4, 5). Most of these studies are conducted in clinical academic centers, which manufacture CAR T products in their own cell production facility. The ability to produce point-of-care CAR T is essential for the development of novel CAR T therapies or the evaluation of CAR T efficacy in additional indications.

Furthermore, on-site production dramatically reduces the turnaround time from leukapheresis to infusion, which is a major obstacle with commercial CAR T cell products and may reduce cost. Since commercial products are centralized manufactured and cryopreserved, the average time from leukapheresis to infusion is typically 30 to 45 days, including shipment, manufacturing, and QC testing for cryopreserved cell products. Thus, rapidly progressive patients may clinically deteriorate during the waiting period (6).

Disease control during CAR T manufacture can be difficult, especially in patients with aggressive disease refractory to conventional therapies (7–9).

In-house production of fresh, non-cryopreserved CAR T products can reduce the turnaround time to only 6 to 10 days, as no shipment and faster sterility QC testing is required, thereby enabling the treatment of patients with most aggressive disease.

In 2016, the Sheba Medical Center initiated a phase 1b/2 study (NCT02772198) with locally produced CD19 CAR T cells for the treatment of CD19 positive malignancies (10). In this study, CAR T cell production was mostly initiated from non-cryopreserved fresh leukapheresis products and patients received non-cryopreserved CAR T cells for immediate infusion.

Point-of-care production provides the flexibility to cryopreserve cells at any stage, including the leukapheresis product, cells during the expansion phase or the infusion product, if clinically indicated.

In a study by Panch et.al., the authors analyzed the effect of cryopreservation on PBMCs and CAR T cell products in 147 patients from six single-center clinical trials. CAR T cells manufactured from cryopreserved or fresh PBMCs yielded a similar transduction efficacy and CD4/CD8 ratio on the day of cell infusions. There was also no impact on the frequency of CAR T cells, CD4 or CD8 cells following cryopreservation of CAR T products (11). It should be noted that the fresh and frozen cells derived from different patients (11, 12).

In another report of BCMA CAR T-cell production, cryopreserved CAR T cells retained their anti-tumor functions in a NOD/SCID mice model, although cytokine secretion was significantly lower compared to fresh CAR T cells (13).

Here we report our experience with 118 CD19 CAR T products. CAR T cells were mainly produced from patients with ALL and non-Hodgkin’s lymphoma (NHL), but also from patients with r/r Richter’s transformation and CD19 positive acute myeloid leukemia (AML) (10, 14–16). We compared the potency and proliferation capacity of CAR T cells initiated from cryopreserved versus fresh leukapheresis products and the anti-tumor reactivity of cryopreserved versus fresh infusion products.

We performed for the first time a broad phenotype analysis, examined the effect of cell recovery on clinical response, analyzed the recovery of CAR T cells, which were frozen during the manufacturing process and compared the phenotype and anti-tumor reactivity of CAR T cells cryopreserved on day 6 and day 10. Importantly, experiments were not only performed in the cohort of 118 patients, but also head-to-head with fresh and cryopreserved cells of the same patient.

## Material and methods

### Patients

This study was designed as a phase 1b/2 trial, approved by the Israeli Ministry of Health and registered at clinicaltrial.gov (NCT02772198). All enrolled patients signed an informed consent. Depending on age, the minimal performance score was 50 on a Lansky or on a Karnofsky scale. Patients with prior CD19 directed therapies were eligible for the study. Lympho-depleting preconditioning was inducted by fludarabine 25 mg/m^2^ for 3 days (2 to 4 days before infusion) and cyclophosphamide 900 mg/m^2^ for 1 day (2 days before infusion), followed by infusion of 1x10^6^ transduced CD19 CAR T cells per kilogram weight. Primary endpoints of the study were production feasibility, patient safety and best overall response rates, documented one to two months after infusion.

### CAR T production

A leukapheresis product was used as starting material for autologous CD19 CAR T cell production. Peripheral blood mononuclear cells (PBMC) were isolated from the apheresis product by a single step density gradient with Ficoll-Hypaque (Lymphocyte Separation Medium, Axis-Shield Diagnostics, Scotland). 400x10e6 PBMC were re-suspended at the concentration of 1x10e6 cells per ml in complete medium (CM), containing 10% human AB serum (Valley, VA, US), 2mM L-Glutamine (Biological Industries, Israel), Pen/Strep (Biological Industries, Israel) in AIM-V medium (Invitrogen, CA, USA). 300 IU/ml interleukin (IL)-2 (Clinigen Healthcare, United Kingdom) and 50 ng/ml anti-CD3 monoclonal antibody OKT-3 (Miltenyi Biotec, Bergisch Gladbach, Germany) were added to the medium.

On day 2, 60x10e6 cells were transduced with the CD19 CAR retroviral vector and the rest of the cells discarded. The retroviral supernatant was generated from the CD19 CAR master cell bank PG13-CD19-CAR-H3 kindly provided by Dr. Steve Rosenberg, NCI (17, 18). In short, a plasmid encoding the CD19 CAR consisting of the mouse stem-cell virus gamma-retroviral backbone engineered to express a single chain variable region moiety (scFv) derived from the mouse anti-CD19 hybridoma, FMC63 (19) fused to intracellular domains from human CD28 and CD3 zeta was used for viral vector production. For transduction, non-tissue culture treated 6-well plates were coated with 10 µg/ml RetroNectin^®^ (Takara Bio Inc, Otsu, Japan) in PBS for 2 hours at room temperature or overnight at 4°C, followed by 30 minutes blocking with 2.5% human albumin (Zenalb20, Bio Products Laboratory) in PBS and washed. Retroviral supernatant was rapidly thawed, diluted 1:1 with AIM-V medium supplemented with 5% human AB serum. Four ml of the diluted vector were added per well of the RetroNectin-coated plates and centrifuged at 2000xg for 0.5 to 2 hours at 32°C. The supernatant was aspirated, and cells were re-suspended in CM medium with 300 IU/ml IL-2. 2.5x10^6^ cells were added to each well (total of 60x10^6^ cells in four 6-well plates), centrifuged for 15 minutes at 1000xg and incubated at 37°C overnight.

On day 3, cells were collected from the 6-well plates. For easy removal of attached cells from the plate surface, 1.5ml 10mM EDTA buffer (Biological Industries, Israel) was added per well and incubated for up to 5 min at 37°C. Detached cells were collected and centrifuged at 2000xg for 10 minutes. The supernatant was aspirated, and cells were re-suspended in CM medium with 300 IU/ml IL-2. 0.5x10^6^ cells/ml and transferred into T175 or GRex100 flasks (20) for expansion. When using GRex100 flasks, CM medium was topped up once to 450 ml on day 6. When using T175 flasks, medium was added every other day to maintain a concentration of 0.5-2.0x10^6^ cells/ml until day 10, the day of infusion.

On the day of infusion, 1x10^6^ CD19 CAR T cells/kg were re-suspended in 0.9% sodium chloride (Baxter, FKE 1323) containing 2.5% human albumin and 300 IU/ml IL-2 and delivered to the patient for immediate infusion.

### Cryopreservation and thawing

If indicated, cells were cryopreserved in Cryostor^®^ cell cryopreservation media-CS10 (Sigma-Aldrich, Israel) at 10-300x10^6^ cells/ml in 1.8ml chilled cryo-vials. The cryo-vials were transferred to Nalgene Cryo 1°C Cooler container and placed in a −80°C freezer overnight. Frozen specimens were transferred to a liquid nitrogen freezer within 24h.

Cryopreserved cells were thawed in a 37°C water bath until single ice crystal remained. The thawed cell suspension was transferred to a 15 ml tube containing 10ml CM medium, centrifuged and washed a second time with CM medium.

### *In vitro* reactivity

To demonstrate *in vitro* anti-tumor reactivity, IFNγ secretion was measured following co-incubation of CD19 CAR T cells with target cells (18, 21–23). Un-transduced T cells served as negative control. The following CD19-expressing target cell lines were used: NALM-6 (acute lymphoid leukemia); Toledo (B cell diffuse large cell lymphoma) and CD19-K562 (K562 cell line transduced to express CD19). The CD19-negative cell line CCRF-CEM (T cell leukemia) served as negative control. All tumor lines were kindly provided by Dr. Steve Rosenberg, NCI.

The co-culture was performed with an effector to target ratio of 1:1 (1×10^5^ each) in a total of 200μl medium overnight at 37°C. Supernatant was collected, if necessary diluted and IFNγ secretion was determined by ELISA (Human IFN- ELISA MAX Deluxe Set, Cat. 430106, BioLegend, San Diego, CA). Measurements were performed in triplicates.

### Flow cytometry

The following antibodies were used for flow cytometry: CD3 (VioBlue; Miltenyi Biotech or Pacific blue and PE; BioLegend), CD4 (FITC or APC-Cy7; BioLegend), CD8 (PE-Cy7 or FITC; clone HIT8a,; BioLegend), CD3/CD19 antibody kit (FITC/PE; BD), CD28 (PerCP-Cy5.5; eBioscience), PD-1 (FITC; clone: EH12.2H7; BioLegend), TIM-3 (FITC; clone F38-2E2; BioLegend), LAG-3 (FITC; clone 11C3C65; BioLegend), CD25 (APC; clone BC96; BioLegend), CD28 (APC; clone CD28.2; BioLegend), CD45RA (APC-Vio770; Miltenyi Biotec or Brilliant Violet; BioLegend) CCR7 (PerCP-Vio770; Miltenyi Biotec or PerCP; BioLegend), CCR2 (APC; Biolegend), CCR4 (PE; Biolegend), CCR5 (Alexa Influenza 488; Biolegend), CXCR2 (PE-Cy7; Biolegend) and CXCR3 (FITC; Biolegend), CD14 (PE; Biolegend), CD64 (FITC; Biolegend), 41BB (APC; Biolegend).

CAR expressing T cells were stained with biotin-labeled polyclonal goat anti-mouse F(ab)_2_ antibody (anti-Fab, Jackson Immunoresearch, West Grove, PA) and streptavidin (APC conjugated; BioLegend). CD3+F(ab)_2_+ cells were defined as CAR T cells. Isotype labeled cells (Jackson Immunoresearch cat. # 015-000-002) and untransduced cells served as negative controls. Alternatively, CAR T cells were identified with CD19 CAR Detection Reagent (Biotin; Miltenyi Biotec) and Anti-Biotin antibody (APC; Miltenyi Biotec).

For further characterization, CAR T and PBMCs were stained with antibodies mentioned above. Cells were washed and re-suspended in cell staining buffer (BioLegend, San Diego, CA), incubated for 30 min with the antibodies on ice, washed and measured using MACSQuant FACS cytometer (Miltenyi Biotec). Samples were analyzed by FlowJo software (FlowJo LLC, Ashland, OR).

## Results

### Patients’ characteristics

Between May 2016, and March 2020, 124 patients with r/r CD19 positive malignancies were enrolled to the trial, including 41 (35%) patients with ALL, 71 (60%) with NHL, four (3%) with Richter’s transformation and two (2%) with acute myeloid leukemia (AML). CAR T cells were manufactured as described in the method section. Six CAR production were discontinued due to clinical deterioration of the patient (n=5) or production failure (n=1), resulting in 118 completed manufacturing processes and treatments. All patients were heavily pretreated. Patient characteristics and clinical results of 99 patients were previously reported (21).

### Fresh versus cryopreserved PBMCs

CAR T cells production was initiated from PBMCs collected by leukapheresis. The stability of the apheresis product was evaluated for three patients during which the apheresis product was stored at room temperature on a rocker. Viability, cell number and morphology were examined, and frequencies of CD3 T cells and 7-AAD positive, dead cells were evaluated by FACS. The stability of the fresh apheresis product was determined as 24 hours (**Supplemental Table 1**).

**Figure 1:**
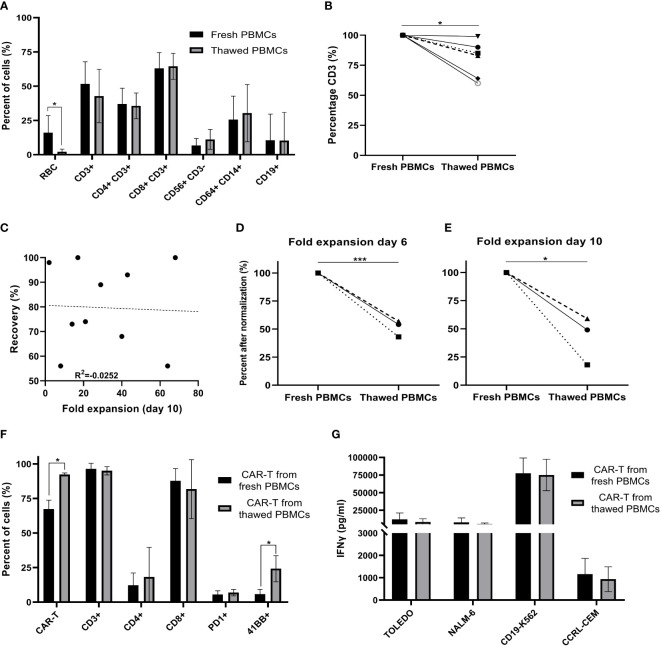
Comparison of fresh versus cryopreserved PBMCs. **(A)** Fresh PBMC and cryopreservation/thawed PBMCs subpopulation composition markers, were analyzed by flow cytometry (RBC; red blood cells) (n=6). **(B)** % CD3+ T cells in fresh PBMCs and thawed PBMCs after normalization (fresh PBMCs = 100%) (n=6). Each symbol represents a single patient’s product. **(C)** Spearman’s rho correlation between the recovery (%) after thawing and the fold expansion achieved by the end of the production process. (n=10). Each dot represents a single patient’s product. **(D, E)** Fold expansion on day 6 and day 10 after normalization (fresh PBMCs = 100%). Average fold expansion ± SD in comparison to the number of cells taken for transduction on day 2 (n=3). Each symbol represents a single patient’s product. **(F)** Phenotype analysis of CAR T cell products on day 10 (n=3). Each symbol represents a single patient’s product. **(G)** CAR T cell potency determined by IFNγ secretion after co-culture with CD19 expressing target cell lines (Toledo, NALM-6 and CD19-K562) and the CD19 negative cell line (CCRL-CEM) (n=3). *p≤.05, ***p≤.0001.

PBMCs were isolated from the apheresis product by density gradient. To determine how cryopreservation affects the subpopulation composition of freshly isolated PBMCs, flow cytometry was performed on six patient samples before cryopreservation and after thawing. Following density gradient purification of fresh PBMCs, 16 ± 11% red blood cells (RBC) were present in the cell suspension, which decreased to 2 ± 2% after cryopreservation (p = .022). Freezing of cells had no impact on the average frequency of CD4+CD3+ helper T cells (p = .500), CD8+CD3+ cytotoxic T cells (p = .149), CD56+CD3- NK cells (p = .085), CD14+CD64+ monocytes (p = .136) and CD19+ B cells (p = .149) (**Figure 1A**). The average percentage of total CD3+ T cells in the starting material decreased from 52 ± 15% in fresh PBMCs to 43 ± 18% in cryopreserved and thawed PBMCs (p = .410). After normalization (fresh PBMC = 100%), the number of CD3+ T cells dropped by 20 ± 14% following thawing (p = .010) (**Figure 1B**), while cryopreservation had no impact on all other subpopulations also after normalization (p ≥.085).

CAR T production for therapy was initiated with fresh PBMCs (n = 108) or cryopreserved PBMCs (n = 10). Cryopreserved PBMCs were stored on average 24 days (range 7 to 45 days) before thawing. Recovery after thawing was 81 ± 16% (range 56 to 97%). There was no correlation between recovery and fold expansion of CAR T obtained by day10 of the production process (R = -.0252; p = .945) (**Figure 1C**).

On day 0, 400x10^6^ PBMCs were stimulated with anti-CD3 antibody in IL-2 containing culture medium. Cryopreserved PBMCs were washed and immediately activated. As anti-CD3 antibody activation supports the expansion of T cells, but deprives CD3 negative cells, the cell number two days after stimulation dropped by an average of 35% (258 ± 177x10^6^). There was no significant difference in cell number on day 2 between fresh and cryopreserved PBMCs as starting material (p = .104) (**Table 1**). Transduction was performed with 60x10^6^ cells on RetroNectin-coated plates overnight and the remaining cells were discarded. The fold expansion on days 6 and 10 compared to day 2 were similar in cultures initiated from fresh and cryopreserved PBMCs (p = .523, p = .133 respectively) (**Table 1**).

**Table 1:** Production and infusion characteristics of CAR T derived from fresh and frozen PBMC.

	**Total Productions** **(n =118)**	**Fresh PBMCs** **(n = 108)**	**Cryopreserved PBMCs** **(n = 10)**	**p value**
**Day 0**				
Cell no. at initiation, (x10e6)	400	400	400	1
**Day 2**				
Cell no. (x10e6)	258 ± 177	266 ± 179	170 ± 129	.104
Cell no. for transduction (x10e6)	60	60	60	1
**Day 6**				
Total cell no. (x10e6)	417 ± 341	411 ± 342	484 ± 328	.523
Fold expansion*	7 ± 6	7 ± 6	8 ± 9	.523
CAR T no. (x10e6)	259 ± 255	253 ± 255	320 ± 246	.432
CAR T cell (%)	57 ± 17	56 ± 17	64 ± 14	.166
**Day 10**				
Total cell no. (x10e6)	1337 ± 1079	1291 ± 1045	1830 ± 1294	.133
Fold expansion*	22 ± 18	22 ± 17	31 ± 22	.133
CAR T cell no. (x10e6)	856 ± 724	817 ± 687	1274 ± 943	.057
**Infusion product**				
CAR T cell no. (%)	66 ± 18	65 ± 19	74 ± 12	.157
CD3+ (%)	94 ± 11	93 ± 12	97 ± 4	.313
CD4+ CD3+ (%)	29 ± 17	29 ± 18	25 ± 5	.448
CD8+ CD3+ (%)	71 ± 17	71 ± 28	75 ± 5	.446
CD4+ CAR T (%)	29 ± 18	29 ± 19	25 ± 5	.496
CD8+ CAR T (%)	71 ± 18	71 ± 19	75 ± 5	.493
**Objective response (n; %)**	84 of 115^#^ (73%)	76 of 105^#^ (72%)	8 of 10 (80%)	.727

*Fold expansion in comparison to the number of cells taken for transduction on day 2 (60x10e6).

3 patients were lost to follow up.

The CAR transduction efficacy on day 6 was on average 57 ± 17% and on day 10 66 ± 18% with no significant differences when using thawed or fresh PBMCs as starting material (p = .166 and p = .157, retrospectively). Also, the content of total CD4 and CD8 T cells, and CD4 and CD8 CAR+ cells were similar on day 10 between the two groups (p ≥.446) (**Table 1**).

Clinical response was determined in 115 of 118 treated patients. Three patients were lost to follow up. 73% (84 of 115) patients achieved an objective response, including 72% (76 of 105) patients, who received a CAR T product initiated from fresh PBMCs and 80% (8 of 10) patients, who’s CAR T product derived from frozen PBMCs (p = .727) (**Table 1**).

Due to major patient-to-patient variations regarding CAR T expansion (range 0.64 to 117-fold) and transduction efficacy (range 14 to 95%), we compared fresh and thawed PBMCs derived of the same apheresis product in three patients head-to-head. Direct comparison revealed that fresh apheresis products yielded a higher fold expansion of cells on day 6 (p ≤.001) and day 10 (p = .01) (**Figures 1D, E**, after normalization (fresh PBMC = 100%) and **Supplemental Figure 1**, absolute numbers**)**. However, the CAR transduction efficacy was significantly higher in infusion products initiated from thawed PBMCs (p = .011) (**Figure 1F**). Thus, the overall number of CAR+ T cells was similar on day 10 (fresh CAR T 1,902 ± 1,495 x10^6^; thawed CAR T 1,261 ± 1,279 x10^6^; p = .668). Further phenotype analysis revealed no impact on the frequency of CD3, CD4, CD8 and PD-1 positive T cells (p ≥.262), but a higher percentage of 4-1BB positive cells in infusion products initiated from cryopreserved PBMCs (p = .034) (**Figure 1F**). Cryopreservation had no impact on cell potency, determined as IFNγ secretion after co-culture with the CD19 expressing target cell lines Toledo, Nalm-6 and CD19-K562 (p ≥.562) (**Figure 1G**). These target cell lines strongly differ in their CD19 antigen density, with CD19-K562 having the highest and Toledo the lowest density (**Supplemental Figure 2**).

### Recovery of CAR T cells cryopreserved during expansion

**Figure 2:**
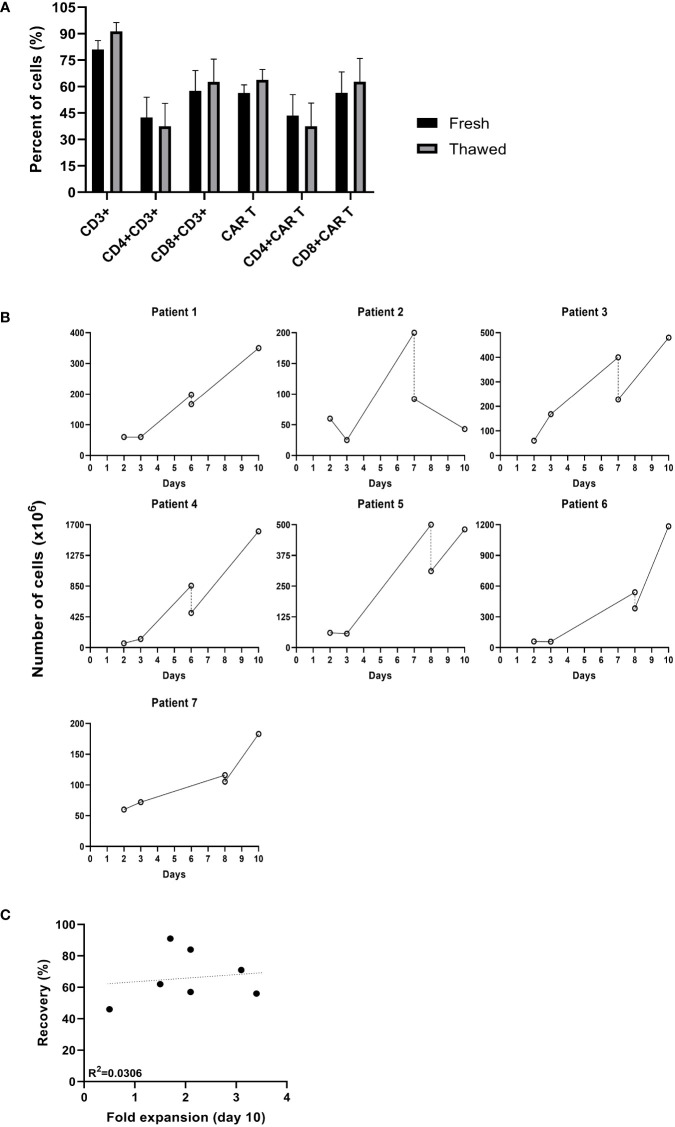
Comparison of cultures that were cryopreserved during the production process. **(A)** Phenotype analysis of fresh CAR T cells before cryopreserved and after thawing determined by flow cytometry (n=7). **(B)** Cell numbers (x10^6^) from transduction to infusion. (–; day of cryopreservation). **(C)** Spearman’s rho correlation between the recovery after thawing and the fold expansion achieved by day 10 of the production process (n=7).

The in-house production of CAR T provides maximal flexibility, often required when treating highly advanced patients. Seven patients clinically deteriorated during the production process, and it was decided to hold the expansion and resume it at a later time point. Cells were cryopreserved between day 6 and 8 of the expansion and stored for an average of 29 days (range 8 to 61 days). The average cell recovery after thawing was 67% (range 56 to 91%). Cryopreservation did not affect the frequency of CAR+ T cells, CD3 T cells or the CD4/CD8 subpopulation composition (p ≥.05) (**Figure 2A**). As shown in **Figure 2B**, in 6 out of 7 (86%) patients, CAR T cryopreserved during the expansion phase continued to proliferate following thawing. Until the time point of cryopreservation, the cells expanded by an average of 7 ± 4 -fold. Following thawing, cells expanded an additional 2.1 ± 0.9 -fold within 2 to 3 days and reached a total cell number of 618 ± 525 x10^6^. There was no correlation between the recovery after thawing and the fold expansion achieved by the end of the production process (R = 0.175, p = .708, **Figure 2C**). In all cases, the target dose of 1x10^6^ CART/kg was reached. Four of the seven patients achieved complete remission.

### Phenotype analysis and anti-tumor reactivity of cryopreserved infusion products

After completion of the manufacturing process, the fresh CAR T product was administered intravenously to the patient. The stability of the non-cryopreserved CAR T cells stored at 5 ± 3°C was evaluated for three infusion products and was determined as 4 hours, based on cell number, viability, morphology, pH, flow cytometry and potency, measured as IFNγ after co-incubation with CD19 positive cell lines (**Supplemental Table 2**).

**Figure 3:**
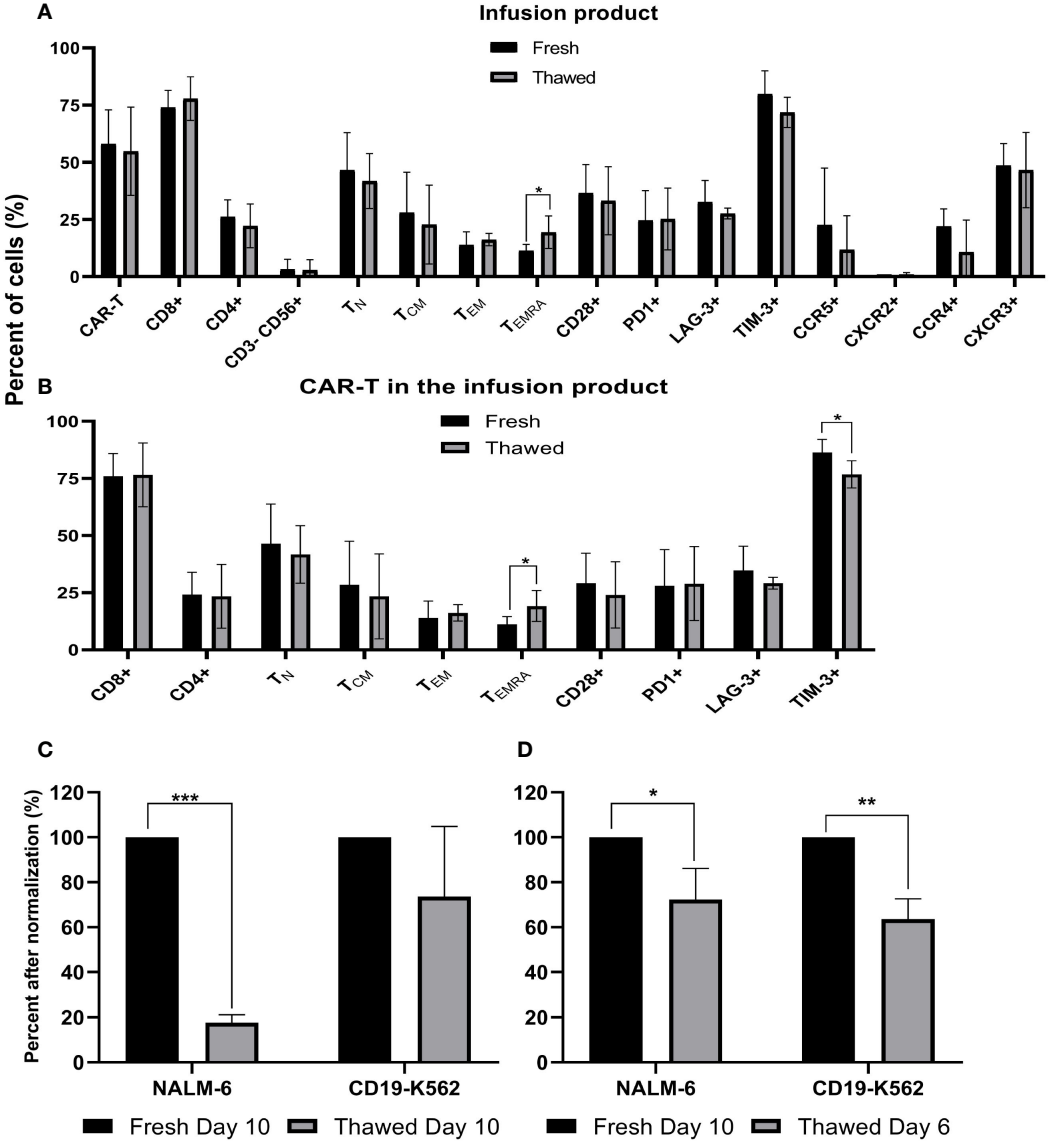
Phenotype analysis and anti-tumor reactivity of cryopreserved infusion products. **(A)** Fresh CAR T infusion products were characterized by flow cytometry before cryopreservation and after thawing (n=5). NK cells, CD3-CD56+; TN (naïve), CD3+ CD45RA+ CCR7+; TCM (central memory), CD3+CD45RA−CCR7+; TEM (effector memory), CD3+CD45RA−CCR7−; TEMRA (effector), CD3+ CD45RA+ CCR7. **(B)** Phenotype analysis of the CAR T infusion products gated on CD3+F(ab)2+ CAR T cells (n=5). **(C+D)** Cell potency determined as IFNγ secretion after co-culture with CD19 expressing target cell lines (NALM-6, CD19-K562). The IFNγ levels are shown after normalization (fresh CAR T, day 10 = 100%) (n=3). **(C)** Fresh and cryopreserved CAR T cells from day 10. **(D)** Fresh CAR T cells from day 10 and cryopreserved CAR T cells from day 6, which were thawed 4 days later. *p≤.05, **p≤.01, ***p≤.001.

Fresh CAR T infusion products of five patients were characterized by flow cytometry before cryopreservation and immediately after thawing. As shown in **Figure 3A**, cryopreservation had no impact on the total percentage of CAR T cells, the CD4/CD8 subpopulation composition (p = .514), expression of the co-stimulatory molecule CD28 (p ≥.701), the co-inhibitory molecules PD-1 (p ≥.808), LAG-3 (p ≥.296), TIM-3 (p ≥.172) and the chemokine receptors CCR5 (p ≥.429), CXCR2 (p ≥.945), CCR4 (p ≥.150) and CXCR3 (p ≥.826). Thawed infusion products contained significantly more CD45RA+CCR7- effector T cells (fresh 11 ± 2%; thawed 19 ± 6; p = .043). The same analysis was performed on the CAR T cell population itself and revealed no differences in the percentage of CD4, CD8, CD28, PD-1, LAG-3 (p ≥.279) (**Figure 3B**). However, thawed CAR T cells in the infusion bag had significantly less TIM-3+ CAR T cells (fresh 86 ± 5%; thawed 77 ± 5; p=.027) and more CD45RA+CCR7- effector CAR T cells (fresh 11 ± 3%; thawed 19 ± 6; p = .047).

To compare the anti-tumor potency (measured as IFNγ secretion following co-incubation with CD19 expressing tumor lines) of fresh versus cryopreserved infusion products, we examined IFNγ secretion after co-culture of CAR T cells with various targets. Initially, we compared fresh infusion products on day 10 (% CAR T cells: 92 ± 1%) and on the same sample 6 weeks after cryopreservation (% CAR T cells: 92 ± 3%). IFNγ secretion after co-culture of CAR T cells with target cells, strongly varied between the three patients (18,000 pg/ml – 182,683 pg/ml), thus analysis was performed after normalization (fresh CAR T cells = 100%) (**Figure 3C**). IFNγ secretion after co-culture with CD19-K562 target cells was similar between fresh and thawed cells (p = .223), but a higher IFNγ secretion was seen when fresh cells were co-cultured with Nalm-6 cells, which have more physiological CD19 expression (p <.001) (**Figure 3C**). Absolute numbers are shown in **Supplemental Figure 3**.

Since several manufacturing protocols cryopreserve CAR T-cells earlier, we also tested cells that were cryopreserved on day 6 of expansion and thawed 4 days later (% CAR T cells: 95 ± 1%) to fresh CAR T-cells from day 10 (% CAR T cells: 92 ± 1%). ELISA assays were performed simultaneously to avoid variations that may occur in the condition of the target cells. Here, IFNγ levels after co-incubation with both CD19-positive target cells were significantly higher with fresh cells from day 10 compared to the thawed cells from day 6 (p= .024; p=.002, respectively) (**Figure 3D**). In all cases, the IFNγ levels after co-incubation with CD19 positive target cells was far above 200pg/ml IFNγ (11,228 pg/ml – 182,683 pg/ml), the acceptance criteria in most clinical trials and at least 1000-fold higher compared to the CD19 negative target cell CCRL-CEM (**Supplemental Figure 4**).

### Comparison of cryopreserved infusion products from day 6 and day 10

In the cohort of 118 patients, the transduction efficacy of fresh cells significantly increased during the expansion phase (day 6, 57 ± 17%; day 10, 66 ± 18%; p ≤.0001) (**Table 1**).

**Figure 4:**
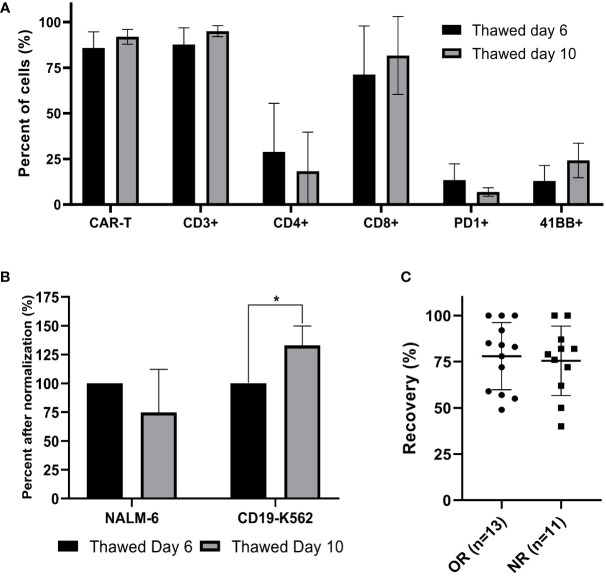
Comparison of cryopreserved infusion products from day 6 and day 10 and correlation between cell recovery and clinical response. **(A)** Thawed CAR T cells from day 6 and day 10 were characterized by flow cytometry (n=3). **(B)** Cell potency of thawed CAR T cells from day 6 and from day 10, determined by IFNγ secretion after co-culture with CD19 expressing target cell lines (NALM-6, CD19-K562). The IFNγ levels are shown after normalization (thawed day 6 = 100%). (n=3); *p≤.01. **(C)** Spearman’s rho correlation between cell recovery (%) and clinical response (OR; objective responder; NR, non-responder). Each dot represents a single patient’s product. Bold lines represent median, with maximum and minimum range. P values were calculated for unpaired t tests.

Cells from three patients were cryopreserved on day 6 and day 10. The thawed cells were analyzed simultaneously. The transduction efficacy in these three samples was insignificantly higher on day 10 compared to day 6 (day 6, 86 ± 7%; day 10, 92 ± 3%; p = .166). As shown in **Figure 4A**, the frequency of CD4, CD8, PD-1 and 4-1BB expressing cells was also similar (p ≥.199). IFNγ levels after normalization was significantly higher after co-incubation of CD19-K562 with CAR T cryopreserved on day 10 compared to day 6 (p= .029), but no differences were noted in IFNγ levels after co-incubation with Nalm-6 (**Figure 4B**, **Supplemental Figure 5**).

### Correlation between cell recovery and clinical response

The recovery of CAR T infusion products was determined in 24 samples (13 objective responders and 11 non-responders). All 24 patients were treated with fresh CAR T cells and frozen samples were stored on average 313 days (range 53 to 935 days) in a nitrogen tank. The recovery was 77 ± 18% (range 40 - 100%). There was no correlation between the cell recovery and clinical response (p = .753) (**Figure 4C**). As shown before, cryopreservation had no impact on the transduction efficacy (p = .294) and the CD4/CD8 subpopulation composition (p= .284).

## Discussion

CAR T cell therapy has shown high response rates in relapsed and refractory patients with hematological diseases. To date there are several approved CD19 CAR T cells for B-cell malignancies, and over 500 clinical trials are currently conducted with CAR T cells in hematological malignancies and solid tumors. Most trials are performed in clinical centers, with many of these centers manufacturing the cells on-site. On-site production reduces costs and shortens the time from apheresis to infusion. Whereas on-site production allows the infusion of both fresh and cryopreserved cells, centralized manufacturing requires cryopreservation, due to the short stability of the cellular product. Currently all FDA approved CAR T products are produced in centralized sites and the cryopreserved drug product shipped to clinical centers for infusion.

Previous reports demonstrated impaired function of cryopreserved NK cells (22) and mesenchymal stromal cells (23) compared to their fresh counterparts. However, Xu et al. could show that cryopreservation had no impact on the transduction efficacy, differentiation status and CD4/CD8 distribution of BCMA CAR T cells (13). Also, similar tumoricidal effects were reported *in vitro* and *in vivo*, although cryopreserved CAR T secreted lower levels of cytokines. Similar cytotoxic activity was also demonstrated in CD19 CAR T cells manufactured on the automated CliniMACS Prodigy device (24). In 2019, Panch et al. analyzed cryopreserved CAR T cell products from 147 patients and concluded that cryopreservation is a viable strategy, since cryopreserved CAR T products yielded a similar transduction efficacy, persistence and CD4/CD8 ratio as their fresh counterparts. The strength of this study is that it encompasses 147 CAR products from six different CAR T protocols, approximately half of the productions were initiated from fresh PBMCs and about half of the patients received a fresh CAR T product. However fresh and frozen cells of the same patients were not directly compared (12).

Here we determined the effect of cryopreservation on PBMCs. There was no impact on the frequency of NK, B-cells, monocytes, CD4 and CD8 T cells, however the frequency of erythrocytes was significantly decreased in the frozen samples after thawing. Although the absolute number of CD3+ T cells was lower in cryopreserved samples, it was sufficient to produce CAR T cells for therapy. Furthermore, there was no correlation between the recovery of PBMCs and the number of CAR T cells obtained by the end of the manufacturing process or the response rate to CAR T therapy.

Panch et al. (11) reported no impact on the fold-expansion and transduction efficacy, when comparing CAR T cells initiated from fresh or frozen PBMCs of different patients. However, by comparing head-to-head PBMCs from the same apheresis product, we could show that fresh PBMCs expanded better, but had a lower transduction efficacy, resulting in a similar total number of CAR T cells by the end of the production process. Also, the *in vitro* anti-tumor reactivity was similar.

Next, we determined for the first time the quality of cells that were cryopreserved during the expansion process, as this might be clinically implicated. Six of 7 CAR T cell products which were frozen on day 6 to 8 of the expansion continued to proliferate after thawing, and all seven CAR T products achieved the required number of cells for infusion. This demonstrates that the production process can be halted and resumed at a later time-point.

The stability of fresh CAR T products is limited, which means that cryopreservation is a necessity for the production at centralized sites. There was no difference between the cell recovery, transduction efficacy and CD4/CD8 frequency in CAR T cells, which were cryopreserved from patients which responded to CAR T cell therapy and non-responders. A broad phenotype analysis performed on fresh CAR T product and their frozen counterparts demonstrated no impact on the frequency of CD4 helper T cells, CD8 cytotoxic T cells, the co-stimulatory molecule CD28, the co-inhibitory molecules PD-1 and LAG-3, and a variety of chemokine receptors. TIM-3 was significantly increased within the CAR T population of fresh infusion products. In general, TIM-3 is considered a negative regulator of innate and adaptive immune responses and plays a role in cancer immunity (25–27). Although TIM-3 is widely found to be associated with acquisition of T cell exhaustion, it was recently shown that a major function of TIM-3 is to enhance T cell activation during viral infection, and that TIM-3 is actually dispensable for the development of T cell exhaustion (28). It was further shown that PD-1+ TIM-3+ CD8+ T cells produce fewer cytokines than PD-1+ TIM-3– CD8+ T cells upon stimulation with cognate antigen, but paradoxically exhibit superior lytic capacity and antitumor reactivity *in vivo* (29, 30). The significance of increased TIM-3 expression in the CAR T cell population of fresh products requires further investigation.

Differentiation status analysis revealed significantly less terminally differentiated effector (TEMRA) CAR T cells in fresh CAR T drug products compared to frozen products. Several studies demonstrate that terminally differentiated effector T cells exhibit shorter persistence and are inferior in achieving long-term immunity after adoptive cell therapy compared with naïve or memory T cells (31, 32). Further studies are required to determine if fresh CAR T cells, with a lower frequency of TEMRA cells, persist longer after therapy than frozen products.

Fresh CAR T infusion products demonstrated increased *in vitro* anti-tumor reactivity, however cryopreserved CAR T cells revealed still high anti-tumor potency and specificity. The cell recovery of cryopreserved CAR T products was similar in responding and non-responding patients.

Cryopreserved infusion products contained the same percentage of CAR T cells as their fresh counterpart and demonstrated very high *in vitro* reactivity, no matter if they were cryopreserved on day 6 or day 10 of the manufacturing process.

One limitation of this study is that variations in the production process may impact the CAR T product quality. Here, CAR T cell production was initiated from PBMCs, which were stimulated with soluble anti-CD3 monoclonal antibody and expanded in an open system. Other studies start from CD4/CD8-enriched T cells with CD3/CD28 bead activation in a closed system. In addition, the cryopreservation process was performed in freezing containers in contrast to controlled-rate freezers, which may affect cell recovery and drug product quality. Furthermore, cytotoxicity assays with different E:T ratios might help to determine minor differences in CAR T potency.

The legitimacy of using cryopreserved CAR T cells is further supported by clinical results with the commercial cryopreserved CAR T product axicabtagene ciloleucel (axi-cel), which contains a CAR T construct similar to the one used in this study (33). In a retrospective analysis with 1,125 Caucasian patients with relapsed or refractory large B-cell lymphoma the objective overall response rate (OOR) was 74%, including 57% complete responses (CR) and the 12-months progression free (PFS) and overall survival (OS) 48% and 65%, respectively. In our clinical trial with fresh CAR T cells in 72 patients with r/r B cell lymphoma the OOR was 63%, including 38% CR, and the median 12-months PFS and OS 40% and 52% respectively (34). Although the clinical results of axi-cel and our CD19 CAR T trial clearly cannot be directly compared, the consistent clinical response rates and survival outcomes with axi-cel in the real-world setting, strengthens the validity of cryopreserved CAR T products for therapy.

To conclude, we show comparability of thawed versus fresh cells PBMCs and CAR T cell products. The use of frozen PBMCs as staring material seems to be a viable choice, as transduction rates are high and the cell phenotype, number and potency by the end of the production process comparable to products initiated from fresh PBMCs. The cryopreservation of CAR T cells during manufacturing, and continuation of the expansion at a later time-point, was feasible and complete responses were achieved. CAR T cells expanded until day 10 demonstrated a significantly higher transduction efficacy compared to CAR T cells from day 6. There was no correlation between the cell recovery of CAR T products and clinical response achieved.

Remission after CAR T cell therapy can be achieved with the use of fresh as well as thawed products, though in preclinical correlates fresh CAR T cells seemed to be superior. As point-of-care CAR T production is gaining increased popularity adding control on the timing of the process and patient care, it may be better to start the manufacturing process with fresh or cryopreserved PBMCs and infuse fresh products.

## Data availability statement

The raw data supporting the conclusions of this article will be made available by the authors, without undue reservation.

## Ethics statement

The studies involving human participants were reviewed and approved by Israeli Ministry of Health and registered at clinicaltrial.gov (NCT02772198). Written informed consent to participate in this study was provided by the participants’ legal guardian/next of kin.

## Author contributions

KB-D and MB contributed conception and design of the study. KB-D, OI, JM, AK, LZ, EJ, AA, RF performed experiments and acquired the data. KB-D wrote the first draft of the manuscript. EJ and MB revised it critically for important intellectual content. All authors contributed to the article and approved the submitted version.

## Acknowledgments

We would like to thank Haya and Nechemia Lemelbaum for their generous support and Abraham Nissani for his technical assistance.

## Conflict of interest

The authors declare that the research was conducted in the absence of any commercial or financial relationships that could be construed as a potential conflict of interest.

## Publisher’s note

All claims expressed in this article are solely those of the authors and do not necessarily represent those of their affiliated organizations, or those of the publisher, the editors and the reviewers. Any product that may be evaluated in this article, or claim that may be made by its manufacturer, is not guaranteed or endorsed by the publisher.
