# Supplementary material for: Impact of cryopreservation on CAR T production and clinical response
Source: Front Oncol. 2022 Oct 6;12:1024362. doi: 10.3389/fonc.2022.1024362 (PMC9582437; doi:10.3389/fonc.2022.1024362)
Supplement: Supplementary file 1 [file Table_1.docx]

**Suppl. Table 1. Stability of the apheresis product.**

|  | **Time point 0** | | | **1 hour** | | | **2 hours** | | | **4 hours** | | | **24 hours** | | |
| --- | --- | --- | --- | --- | --- | --- | --- | --- | --- | --- | --- | --- | --- | --- | --- |
|  | Pt. # 1 | Pt. # 2 | Pt. # 3 | Pt. # 1 | Pt. # 2 | Pt. # 3 | Pt. # 1 | Pt. # 2 | Pt. # 3 | Pt. # 1 | Pt. # 2 | Pt. # 3 | Pt. # 1 | Pt. # 2 | Pt. # 3 |
| **Total cell no**.  (x10e6) | 181 ± 0.40 | 141 ± 0.26 | 65 ± 1.1 | 181 ± 0.04 | 137 ± 0.20 | 59.2 ± 0.6 | 198 ± 0.14 | 128 ± 0.24 | 65 ± 0.5 | 188 ± 0.15 | 153 ± 0.06 | 55 ± 1.2 | 195 ± 0.31 | 157 ± 0.30 | 57.5 ± 1.1 |
| **Viability**, (%) | 98 ± 1 | 99 ± 1 | 97 ± 2 | 99 ± 1 | 98 ± 2 | 98 ± 2 | 100 ± 1 | 99 ± 1 | 100 ± 1 | 97 ± 2 | 98 ± 1 | 97 ± 1 | 95 ± 2 | 97 ± 3 | 99 ± 1 |
| **CD3+**, (%) | 33.0 ± 3.0 | 35.7 ± 0.6 | 44.5 ± 0.6 | 36.8 ± 0.3 | 37.6 ± 0.8 | 43.0 ± 0.1 | 39.0 ± 0.3 | 40.1 ± 0.3 | 42.3 ± 0.5 | 30.5 ± 0.5 | 34.1 ± 0.4 | 48.4 ± 0.4 | 34.9 ± 0.4 | 35.5 ± 0.3 | 54.5 ± 0.4 |
| **7-AAD**, (%) | 3.4 ± 1.1 | 2.8 ± 1.4 | 2.7 ± 0.5 | 5.1 ± 0.5 | 3.1 ± 0.5 | 4.0 ± 0.5 | 5.9 ± 0.3 | 3.8 ± 0.8 | 4.3 ± 0.4 | 2.8 ± 1.1 | 1.9 ± 0.1 | 4.2 ± 0.1 | 6.7 ± 0.2 | 4.9 ± 1.3 | 3.8 ± 0.2 |

(Average ± SD)**.** Apheresis products from three patients (Pt.) were tested for total cell number, viability and analyzed by flow cytometry for CD3 and 7-AAD
