# Supplementary material for: Impact of cryopreservation on CAR T production and clinical response
Source: Front Oncol. 2022 Oct 6;12:1024362. doi: 10.3389/fonc.2022.1024362 (PMC9582437; doi:10.3389/fonc.2022.1024362)
Supplement: Supplementary file 2 [file Table_2.docx]

**Suppl. Table 2.** **Stability of final CD19-CAR product**

Mean ± SD. Fresh, non-cryopreserved CAR T infusion products from three patients were tested for total cell number, viability and analyzed by flow cytometry for CD3, CAR T and 7-AAD.

|  | **Baseline** | | | **4 hours** | | | **24 hours** | | |
| --- | --- | --- | --- | --- | --- | --- | --- | --- | --- |
|  | **Pt. # 1** | **Pt. # 2** | **Pt. # 3** | **Pt. # 1** | **Pt. # 2** | **Pt. # 3** | **Pt. # 1** | **Pt. # 2** | **Pt. # 3** |
| **Total cell no**. (x10e6) | 1.06 ± 0.05 | 120  ± 0 | 133  ± 4.7 | 1.13  ± 0.09 | 133  ± 9.4 | 127  ± 4.7 | 1.13  ± 0.04 | 99  ± 7.7 | 101  ± 6.3 |
| **Viability**, (%) | 94 ± 2 | 97 ± 0 | 94 ± 2 | 98 ± 1 | 96 ± 1 | 91 ± 2 | 94 ± 1 | 68 ± 6 | 63 ± 8 |
| **CAR-T cells,** (%) | 74 ± 0.2 | 44.3 ± 0.4 | 65.8 ± 0.3 | 74 ± 0.3 | 46.0 ± 0.2 | 71.2 ± 0.2 | *ND | *ND | *ND |
| **7-AAD,** (%) | 11 ± 3.1 | 5.0 ± 0.1 | 12.4 ± 0.4 | 8 ± 0.3 | 5.2 ± 0.2 | 12.7 ± 1.1 | 28 ± 0.2 | 23.1 ± 1.7 | 29.3 ± 0.3 |
| **CD8+,** (%) | 51 ± 0.3 | 62.8 ± 0.3 | 68.0 ± 0.2 | 53 ± 0.4 | 62.9 ± 0.1 | 68.2 ± 0.5 | ND | ND | ND |
| **K562- CD19**  (pg/ml IFNγ) | 114,776 ± 6,035 | 236,678 ± 1,925 | 165,530 ± 19,171 | 116,321 ± 3,608 | 270,122 ± 10,007 | 152,218 ± 15,562 | ND | ND | ND |
| **Nalm-6**  (pg/ml IFNγ) | 37,868 ± 3,982 | 88,233 ± 5,804 | 31,463 ± 6,500 | 31,115 ± 780 | 70,162 ± 4,949 | 22,287 ± 1,921 | ND | ND | ND |
| **pH** | 6.8 ± 0.03 | 6.7 ± 0.01 | 6.7 ± 0.05 | 6.9 ± 0.02 | 7.0 ± 0.03 | 6.9 ± 0.04 | 6.8 ± 0.04 | 6.9 ± 0.08 | 6.8 ± 0.07 |
| **Morphology** | Round, shiny | Round, shiny | Round, shiny | Round, shiny | Round, shiny | Round, shiny | Smaller in size | Smaller in size | Smaller in size |

CAR T potency was determined by IFNγ secretion following co-incubation with the CD19 positive cell lines Nalm-6 and K562-CD19. ND = not determined due to decline in cell viability and number.
