# Supplementary material for: Impact of cryopreservation on CAR T production and clinical response
Source: Front Oncol. 2022 Oct 6;12:1024362. doi: 10.3389/fonc.2022.1024362 (PMC9582437; doi:10.3389/fonc.2022.1024362)
Supplement: Supplementary file 3 [file Presentation_1.pptx]

## Slide 1
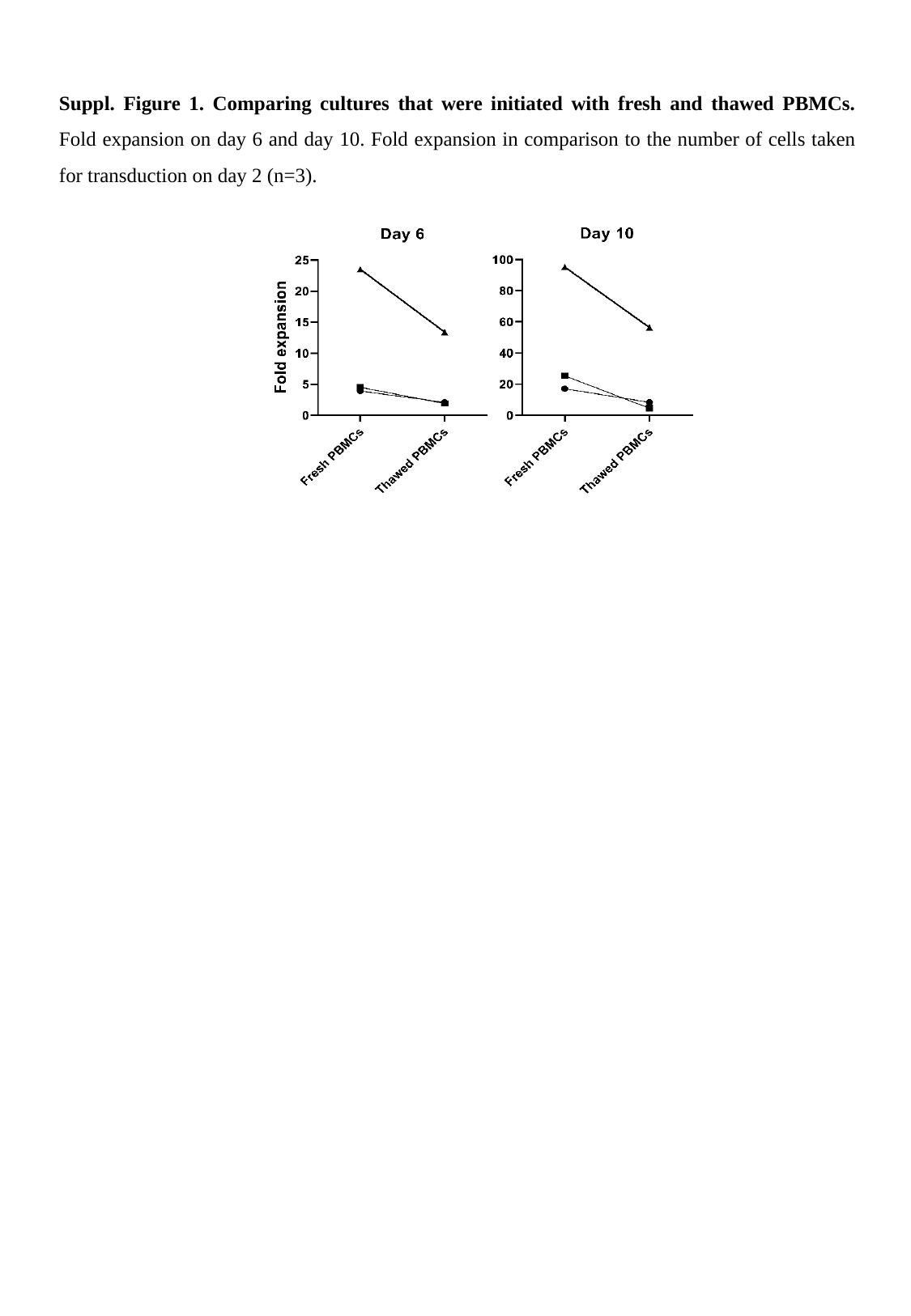

Suppl. Figure 1. Comparing cultures that were initiated with fresh and thawed PBMCs. Fold expansion on day 6 and day 10. Fold expansion in comparison to the number of cells taken for transduction on day 2 (n=3).
