# Supplementary figures and images for: Impact of cryopreservation on CAR T production and clinical response
Source: Front Oncol. 2022 Oct 6;12:1024362. doi: 10.3389/fonc.2022.1024362 (PMC9582437; doi:10.3389/fonc.2022.1024362)

## Slide 1
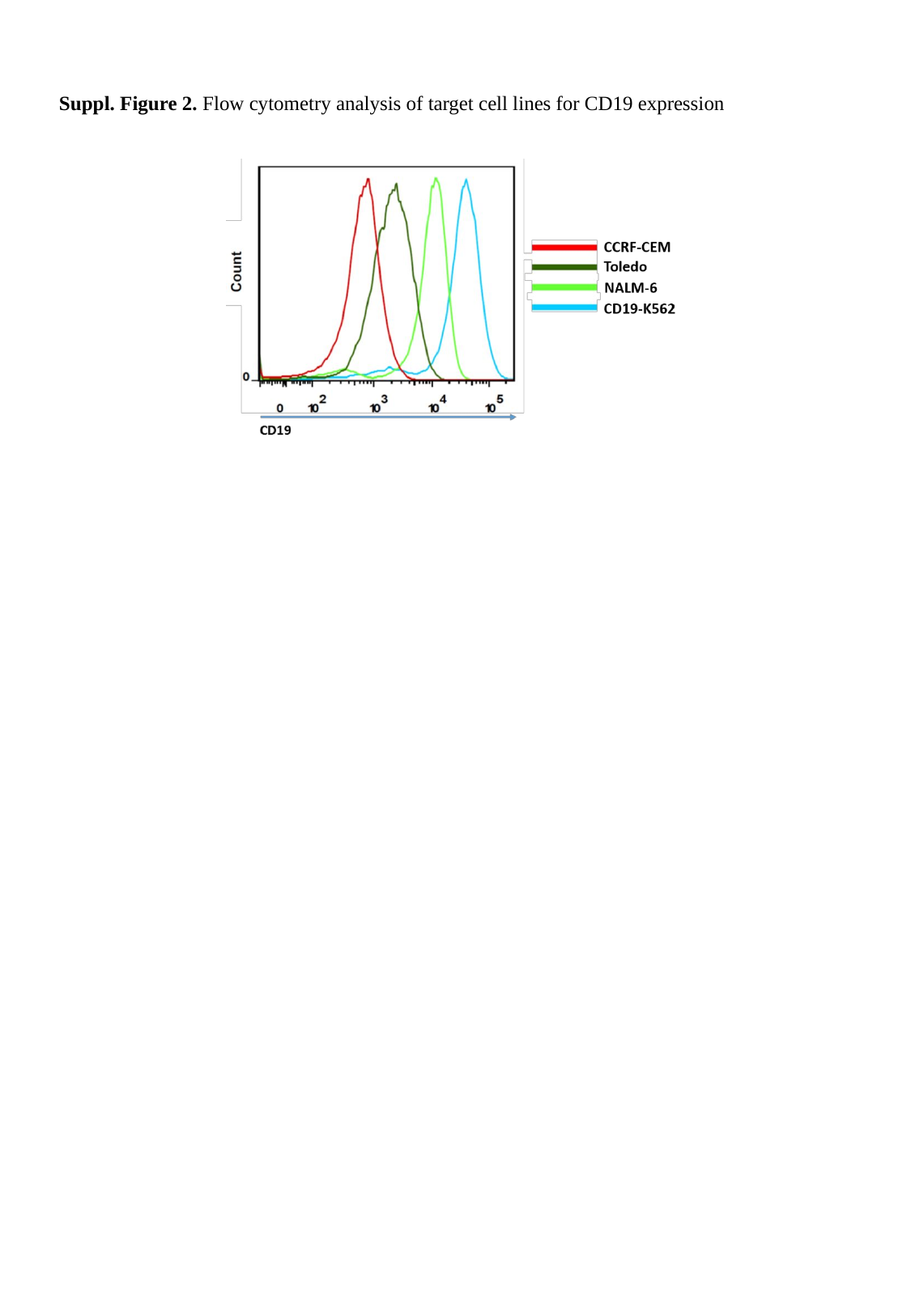

Suppl. Figure 2. Flow cytometry analysis of target cell lines for CD19 expression

Supplement: Supplementary file 4 [file Presentation_2.pptx]
