# Supplementary material for: Impact of cryopreservation on CAR T production and clinical response
Source: Front Oncol. 2022 Oct 6;12:1024362. doi: 10.3389/fonc.2022.1024362 (PMC9582437; doi:10.3389/fonc.2022.1024362)
Supplement: Supplementary file 5 [file Presentation_3.pptx]

## Slide 1
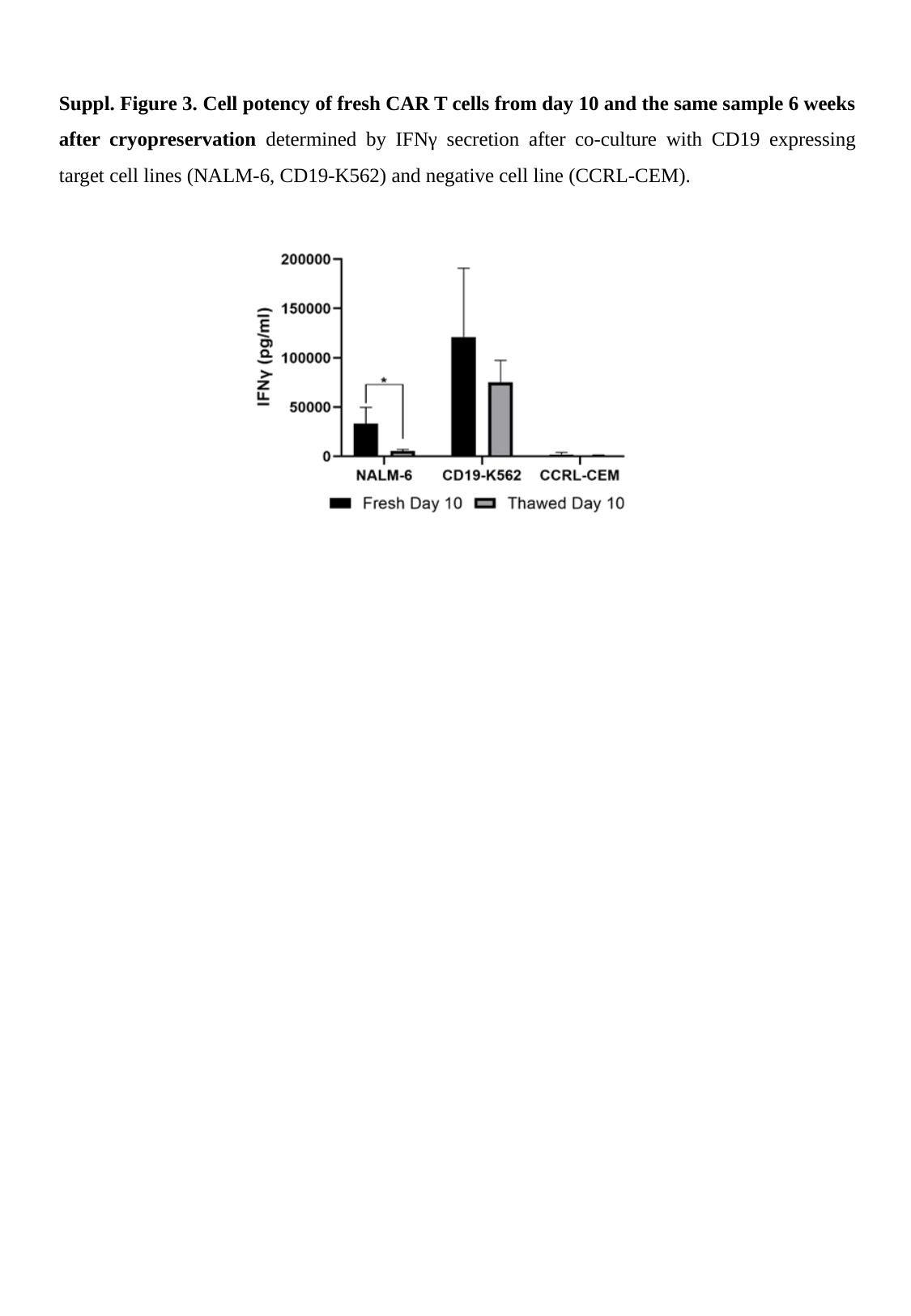

Suppl. Figure 3. Cell potency of fresh CAR T cells from day 10 and the same sample 6 weeks after cryopreservation determined by IFNγ secretion after co-culture with CD19 expressing target cell lines (NALM-6, CD19-K562) and negative cell line (CCRL-CEM).
